# Unraveling genetic predisposition and oxidative stress in vitiligo development and the role of artificial intelligence (AI) in diagnosis and management

**DOI:** 10.5937/jomb0-56661

**Published:** 2025-07-04

**Authors:** Hristina Kocić, Torello Lotti, Tatjana Jevtović-Stoimenov, Uwe Wollina, Yan Valle, Stevo Lukić, Aleksandra Klisić

**Affiliations:** 1 University Clinical Center Niš, Clinic for Dermatology, Niš; 2 University of Niš, Medical Faculty, Niš; 3 Università degli Studi Guglielmo Marconi, Dipartimento di Scienze dela Comunicazione, Rome, Italy; 4 University of Niš, Medical Faculty, Department of Biochemistry, Niš; 5 Academic Teaching Hospital, Municipal Hospital Dresden, Department of Dermatology and Allergology, Dresden, Germany; 6 Vitiligo Research Foundation, New York, USA; 7 University of Niš, Faculty of Medicine, Neurology Department, Niš; 8 University of Montenegro, Faculty of Medicine, Podgorica, Montenegro; 9 Primary Health Care Center, Center for Laboratory Diagnostics, Podgorica, Montenegro

**Keywords:** vitiligo, artificial intelligence (AI), single nucleotide polymorphisms (SNP), melanin, oxidative stress, antioxidative enzymes, vitiligo, veštačka inteligencija (AI), polimorfizam nukleotida (SNP), melanin, oksidativni stres, antioksidativni enzimi

## Abstract

Vitiligo is an autoimmune disorder with a complex genetic and epigenetic aetiology, characterised by progressive skin depigmentation. Recent advancements in artificial intelligence (AI) have greatly impacted the understanding, diagnosis, and treatment of vitiligo. The genetic basis of vitiligo is linked to multiple single nucleotide polymorphisms (SNPs) in genes associated with immune function, apoptosis, and melanogenesis, necessitating the integration of AI for more efficient diagnostic tools and personalised therapies. Genome-wide association studies (GWAS) have identified approximately 50 vitiligo-susceptibility genes, including PTPN1, PTPN22, NLRP1, FASLG, and TYR. These genes influence the immune response and melanocyte function, with the transcription factor Nuclear Factor kappa B (NF-kB), playing a central role in inflammatory responses and redox signaling induced by oxidative stress, in conjunction with antioxidant enzymes such as GPx, GST, SOD, and CAT. AI technologies offer a promising avenue for diagnosing vitiligo by combining genetic, clinical, and imaging data, allowing for more accurate classification and personalised treatment strategies. By analysing vast datasets, AI algorithms can identify patterns within complex genetic markers and clinical features, facilitating earlier and more precise detection of vitiligo. Furthermore, AI-driven approaches can optimise therapeutic monitoring, enabling real-time treatment efficacy and disease progression assessment. Integrating AI in vitiligo genetic diagnostics can revolutionise the monitoring of the disorder, improving patient outcomes through personalised, data-driven interventions.

## Abstract (sr)

Vitiligo predstavlja autoimunsko oboljenje kompleksne genske i epigenetske etiologije, koje je praćeno progresivnom depigmentacijom kože. Nedavna dostignuća u oblasti veštačke inteligencije (AI) značajno su uticala na dijagnostiku i lečenje vitiliga. Genetska osnova vitiliga povezana je sa brojnim genskim polimorfizmima (SNP) na genima koji su povezani sa imunskom funkcijom, apoptozom i melanogeneziom, što zahteva integraciju AI za efikasniju dijagnostiku i personalizovanu terapiju. Genomske studije (Genome-wide association studies GWAS) identifikovale su mutacije na blizu 50 gena, uključujući između ostalih, PTPN1, PTPN22, NLRP1, FASLG i TYR. Ovi geni utiču na imunski odgovor i funkciju melanocita, pri čemu transkripcioni faktor Nuclear Factor kappa B (NF-kB) igra ključnu ulogu u inflamatornom odgovoru i redoks signalizaciji indukovanoj oksidativnim stresom, zajedno sa antioksidativnim enzimima GPx, GST, SOD i CAT. Tehnologije veštačke inteligencije pomažu u dijagnostikovanju vitiliga kombinovanjem podataka genetskih baza, kliničkih nalaza i imidžing tehnika, što omogućava precizniju klasifikaciju i personalizovanu strategiju lečenja. Analizom velikih skupova podataka, AI algoritmi mogu identifikovati obrasce u složenim genetskim markerima i kliničkim karakteristikama, olakšavajući raniju i precizniju detekciju vitiliga. Pored toga, pristupi zasnovani na AI mogu optimizovati praćenje terapije, omogućavajući procenu efikasnosti tretmana i napredovanja bolesti u realnom vremenu. Integracija AI u istraživanje genetske osnove oboljenja poseduje potencijal da revolucionizuje način na koji se bolest može pratiti, poboljšavajući ishod lečenja pacijenata kroz personalizovane intervencije na bazi svih dostupnih podataka.

## Introduction

Vitiligo is a chronic skin disorder characterised by the progressive loss of melanocytes, leading to depigmented patches on the skin [1] [2]. Comparatively, it is the most prevalent depigmenting disorder, which affects both men and women equally, with a global frequency of ∼0.06–8.8%. The three nations with the most significant recorded prevalences are Mexico (2.6–4%), Japan, and India (8.8%) [3]. Incidence rates in separate research demonstrated an upward trend across the periods under investigation. It was estimated that 0.36% of people worldwide had vitiligo throughout their lifetime [4]. The pathophysiology of vitiligo is complex and involves multiple interrelated mechanisms, including genetic predisposition, autoimmune response, oxidative stress, and environmental triggers. Identifying any internal or external factors (genetic, physical trauma, or metabolic) that may be causing the condition is critical. The genetic basis of vitiligo involves complex interactions between genetic polymorphisms and environmental factors, necessitating advanced technological approaches for efficient diagnosis and personalised therapeutic strategies. Vitiligo is a hereditary condition that has a polygenic pattern and multifactorial inheritance. Roughly fifty genes have been linked to vitiligo by genome-wide association studies. The vast majority of these genes are immune genes, indicating that the immune system plays a crucial role in the pathophysiology of vitiligo [5] [6]. Various environmental factors can trigger or exacerbate vitiligo in genetically susceptible individuals. UV radiation is a well-documented trigger that can lead to sunburn, releasing inflammatory mediators. Other potential triggers include stress, skin trauma, and exposure to certain chemicals. Their interactions are crucial for developing targeted therapies and preventive strategies for vitiligo [7].

Artificial intelligence (AI) is revolutionising the fields of genetics, diagnostics, and therapy monitoring in dermatology, specifically in vitiligo. AI has emerged as a transformative tool in addressing these challenges through its ability to process vast datasets, identify subtle patterns, and predict outcomes in genetic diagnostics and therapy monitoring. AI can combine data from multiple sources, identify key characteristics for differentiating between vitiligo subtypes, and develop prediction algorithms for vitiligo diagnosis [8]. AI can also substantially improve the efficacy of monitoring therapy and the personalisation of treatment plans for individuals with vitiligo [9].

### Clinical presentations of vitiligo

Understanding the clinical characteristics and progression of vitiligo is essential for diagnosis, management, and addressing the psychosocial aspects of the condition. The clinical manifestation of vitiligo varies among individuals, with several presentations based on the extent and distribution of the affected areas. Vitiligo typically progresses through several stages, such as the initial stage, when it often begins with small, hypopigmented spots that may go unnoticed. These spots can appear on sun-exposed areas, such as the face, hands, and extremities; in the progressive stage, the spots may enlarge and multiply, leading to more extensive areas of depigmentation. The rate of progression can be variable; some individuals experience rapid spreading, while others may have stable periods during which no new lesions develop.

In some cases, particularly with treatment, patients may experience partial repigmentation, where melanocytes gradually return to the affected areas [10]. Nonsegmental vitiligo includes the following: i) generalised vitiligo, formerly known as vitiligo vulgaris; it usually affects both hands and feet and is often bilateral. The conditions that fall under this category are ii) vitiligo acrofacialis, which affects the face, head, hands, and feet; iii) vitiligo universalis, which affects 80–90% of the body; iv) mucosal vitiligo, which is followed by the depigmentation of the oral and genital mucosae; v) mixed vitiligo, which is the simultaneous presentation of segmental and nonsegmental vitiligo; and vi) the uncommon variations, such as minor vitiligo, follicular vitiligo, and vitiligo punctate. Nonsegmental vitiligo is the most common type, accounting for about 90% of cases. Segmental vitiligo can be mono segmental, bisegmental, or plurisegmental, and it manifests unilaterally as an asymmetric distribution of macules and patches. Unclassified vitiligo can present as either one of two symptoms: (1) focal vitiligo or (2) involvement of a single mucosal location affecting the mucosa of the mouth or vagina. Following trauma, the isomorphic response is exemplified by the Koebner phenomenon. Patients with vitiligo may experience various associated features, such as the premature greying of hair, since depigmentation can also affect hair follicles in areas adjacent to depigmented skin. Some patients may develop ocular manifestations, including iris depigmentation, which can increase the risk of certain eye conditions [10] [11] [12].

The model put forth by Hillmer et al. [13] suggests that using AI could assist in vitiligo diagnosis and estimation of severity score, automatically determining the extent of depigmentation as a percentage share of the afflicted lesion. It is possible to make a virtual vitiligo mask by entering original clinical photos of the face into the body scan system. The system generates training and testing data, extracts faces from clinical images, and produces a vitiligo mask. In clinical practice, this percentage’s change over time could be used to determine if therapies had a beneficial or negative effect on patients during follow-up [14] [15].

## Discussion

### The role of oxidative stress in the pathogenesis of vitiligo

Current research focuses on the roles of redox-sensitive components and antioxidant enzymes in the vitiligo development process. Oxidative stress plays a crucial role in the pathogenesis of vitiligo. The crucial understanding that the amino acid tyrosine is also the source of superoxide anion radical during melanin synthesis means that adequate antioxidant protection is required concurrently with melanin synthesis to prevent free radicals from destroying the melanocyte [16] [17] [18]. Reactive oxygen species (ROS) generation may result from environmental factors, such as UV exposure, and can also be produced during inflammatory responses. This oxidative damage directly harms melanocytes and may alter the microenvironment, making it more conducive to immune attacks. Melanocytes, the cells responsible for melanin production, are particularly vulnerable to oxidative stress. These cells contain high levels of tyrosinase and other enzymes involved in melanin synthesis, which can generate ROS during the melanin production. Elevated levels of ROS can damage melanocytes, leading to their apoptosis or dysfunction [17] [18]. The first line of defence against ROS is glutathione (GSH).

An imbalance between antioxidants and pro-oxidants arises from the inactivation of glutathione reductase 4 (GPX4) due to GSH depletion. GPX, catalase (CAT), methionine sulfoxide reductase (MSR) A and B, and other antioxidant enzymes have downregulated expression in vitiligo skin lesions. The Nrf2 signalling system initially responds to oxidative stress by acting as an antioxidant. Increased ROS activation can activate the transcription factor Nrf2, which translocates to nuclei to control the expression of certain detoxifying and antioxidant gene groups. The cytoplasmic Keap1-Nrf2 complex is normally in an inhibitory state. Upon oxidative stress, the Keap1-Nrf2 complex disintegrates. The liberated Nrf2 is then taken up by the nucleus and attaches itself to the antioxidant-response elements (AREs) of gene promoters to initiate the transcription of many antioxidants and detoxifying enzymes [19] [20].

AI represents a powerful tool in advancing the relationship between oxidative stress and vitiligo. AI may enable the identification of oxidative stress biomarkers, the prediction of disease progression, the discovery of novel therapeutic targets, and the development of personalised treatment strategies. Incorporating AI into the study of oxidative stress offers deeper insights into vitiligo pathophysiology, leading to more effective and individualised therapeutic approaches that address this multifaceted autoimmune disorder. AI can also integrate genomic, transcriptomic, proteomic, and metabolomic data related to oxidative stress markers and associated signalling pathways, together with high-resolution imaging techniques to visualise oxidative damage at the cellular or tissue level. By synthesising these diverse datasets, AI models can uncover some unrecognised relationships between oxidative stress and vitiligo, including identifying key biomarkers for early diagnosis or prognosis.

### Genetic predisposition to vitiligo

**Figure 1:**
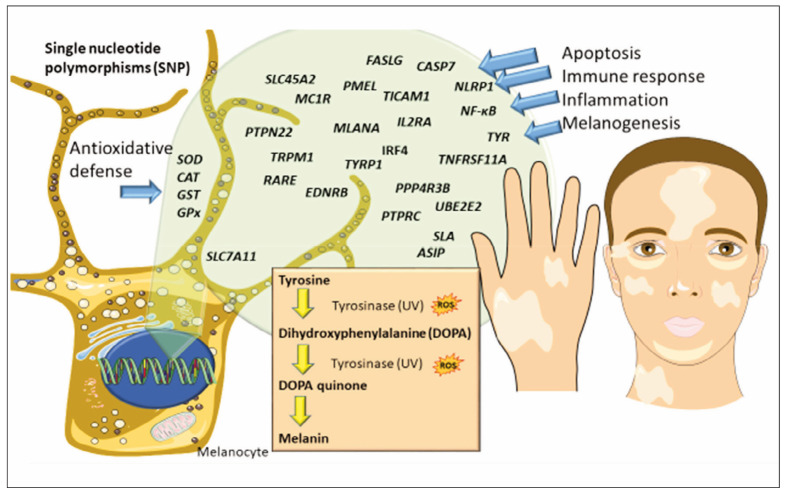
Single nucleotide polymorphisms (SNPs) of genes with a significant role in the susceptibility to vitiligo.

Genetic factors play a significant role in the susceptibility to vitiligo. Several studies have identified various single nucleotide polymorphisms (SNPs) associated with vitiligo, particularly in immune function and pigmentation genes. A multifactorial and polygenic inheritance is assumed in about 50% of cases (range between 46% and 72%). The concordance of vitiligo was found to be 23% in monozygotic twins, while in first-degree relatives of the probands, it was about 7% [21]. The genome-wide association studies (GWAS) have shown that over 20–50 genes may have an impact on the development of vitiligo, known as vitiligo-susceptibility genes, which have a role in apoptosis, immune response, ubiquitination, or melanogenesis (Figure 1). Among the reported are *RARE* (arginine-glutamic acid dipeptide), *PTPN1* (tyrosine-protein phosphatase non-receptor type 1), *PTPN22* (protein tyrosine phosphatase, non-receptor type), *PTPRC* (protein tyrosine phosphatase, receptor type C), *PPP4R3B* (protein phosphatase 4 regulatory subunits 3B), PPP3CA (serine/threonine-protein phosphatase 2B catalytic subunit alpha isoform), *FASLG* (FAS ligand), *UBE2E2* (ubiquitin-conjugating enzyme E2), *IRF4* (interferon regulatory factor 4), *BACH2* (BTB domain and CNC homolog 2), *SLA* (Src-like-adapter), *IL2RA* (interleukin-2 receptor subunit alpha), *ZMIZ1* (zinc finger MIZ domain-containing protein 1), *CASP7* (caspase-7), *TYR* (tyrosinase), *PMEL* (pre-melanosome protein PMEL), *TICAM1* (TIR domain-containing adapter molecule 1), *MC1R* (melanocortin 1 receptor), Solute carrier family 7 member A (SLC7A) and *TNFRSF11A* (tumour necrosis factor receptor superfamily member 11A) [22] [23] [24] [25] [26] [27]. Polymorphisms of *MLANA* (melan-A, melanoma antigen recognised by T cells 1 or MART-1), *TYRP1* (tyrosinase-related protein 1), *PMEL* (premelanosome protein), *OCA2* (P protein), *SLC45A2* (solute carrier family 45 member 2), *GPR143* (G protein-coupled receptor 143), *DCT* (Dopachrome Tautomerase), *TRPM1* (transient receptor potential cation channel subfamily M member 1) and *EDNRB* (endothelin receptor type B) have been reported as well [28].

Polymorphisms in the *NLRP1* gene significantly contribute to the pathogenesis of vitiligo by promoting inflammatory responses and autoimmune processes that damage melanocytes. The NOD-Like Receptor Family Pyrin Domain Containing 1 (NLRP1) pattern recognition receptor, which is encoded by the *NLRP1* gene, is involved in forming inflammasomes and intracellular pathogen recognition. The primary function of human NLRP1 is to activate three caspases. Human NLRP1 oligomerises upon activation by specific PAMPs, and its N-terminal PYD then binds adapter protein ASC. An inflammasome is a multiprotein complex created when ASC binds pro-caspase-1. NLRP1 can bind both pro-caspase-1 and caspase-5. These caspase family members induce apoptosis by activating IL-1 and IL-18. By binding to the patched receptor complex through the adaptor protein DRAL, NLRP1 can activate caspase-9. Activation of NLRP1 can lead to the secretion of pro-inflammatory cytokines like IL-1 and IL-18, which may contribute to the local inflammatory environment in the skin. In the context of vitiligo, aberrant activation of the NLRP1 inflammasome may trigger an autoimmune response against melanocytes. This is supported by findings that show increased expression of NLRP1 in skin lesions of vitiligo patients, indicating its potential role in the pathophysiological mechanism. Dysregulation of these cytokines can result in an immune-mediated attack on melanocytes, facilitating the apoptosis of melanocytes. The interplay between *NLRP1* gene polymorphisms and other genetic predispositions may also influence the severity and progression of vitiligo. Genetic studies have indicated that *NLRP1* may work in concert with other immune-related genes, suggesting a multifactorial model of susceptibility where multiple pathways converge to affect melanocyte viability [29] [30] [31]. For instance, the SNP rs2670660 and rs6502867 have been implicated in increased susceptibility to vitiligo. In the *NLRP1* rs6502867 polymorphism, they were the CT and CC genotypes, while a higher frequency of homozygous GG and heterozygous AG genotypes was reported as the rs2670660 polymorphism [32] [12] [33] [34]. The CT genotype of the *PTPN22* rs2476601 polymorphism is more frequent in vitiligo patients; in the case of the *NLRP1* rs2670660 polymorphism it was the AG genotype, and in the *TYR* rs1393350 polymorphism, it was the AG genotype [32] [35] [24]. Lymphoid-specific tyrosine phosphatase (LYP) is encoded by the *PTPN22* gene found on chromosome 1. The rs2476601 polymorphism has been linked in numerous studies to an increased risk of autoimmune disorders, including vitiligo.

Recent research has focused on how polymorphisms in the *TYR* gene may contribute to the susceptibility and development of vitiligo. The *TYR* gene encodes the enzyme tyrosinase, which is essential for converting the amino acid tyrosine to melanin. This enzymatic process is vital for normal skin, hair, and eye pigmentation. Polymorphisms in the *TYR* (tyrosinase) gene significantly impact the risk and development of vitiligo by affecting melanogenesis, oxidative stress, and immune response. It has been reported that tyrosinase may be an antigen for autoantibodies detected in vitiligo. In terms of SNPs, two SNPs associated with the *TYR* gene area were found (rs1847134 and rs1393350). In the *TYR* gene, the rs1393350 polymorphism has been reported to be the AG genotype [24]. It has been shown that whilst the homozygous GG genotype lowers the risk, the homozygous AA genotype predisposes to vitiligo [36] [37] [29]. The rs1126809 SNP has been linked to altered tyrosinase activity, potentially leading to insufficient melanin production and greater vulnerability of melanocytes to autoimmune attack. Polymorphisms in the *TYR* gene may also influence the production of ROS. Tyrosinase is involved in generating ROS during melanin synthesis. Dysregulated *TYR* expression due to genetic variants can enhance oxidative stress in melanocytes, further contributing to their destruction and the progression of vitiligo. Diagnosis of *TYR* gene polymorphisms in vitiligo may inform personalised treatment strategies. Targeting the molecular pathways affected by these polymorphisms could lead to novel therapies aimed at restoring melanocyte function or modulating immune responses.

The *ASIP* gene, which encodes the Agouti Signaling Protein, regulates melanocyte differentiation and melanin production. ASIP is a melanocyte-stimulating hormone (MSH) receptor antagonist, inhibiting melanogenesis. This balance is critical for maintaining normal skin pigmentation and influencing the

phenotypic expression of skin colour [35]. Several studies have identified specific ASIP gene SNPs associated with an increased risk of vitiligo. SNP rs1393350 has been shown to influence ASIP expression levels, which may disrupt the balance of signalling pathways that regulate melanocyte survival and function. Dysregulation of ASIP due to genetic variants can result in an altered immune microenvironment, promoting autoimmune responses against melanocytes. Inflammatory cytokines may further influence ASIP expression, creating a feedback loop that exacerbates melanocyte damage. Polymorphisms in the *ASIP* gene may also impact the oxidative stress response in melanocytes. Variants that alter ASIP signalling could enhance the production of ROS, contributing to oxidative stress and subsequent melanocyte apoptosis. This mechanism is particularly relevant in the context of vitiligo, where oxidative stress is known to play a critical role in melanocyte loss. Targeting the signaling pathways influenced by ASIP or modifying ASIP expression may provide therapeutic strategies to protect melanocytes and restore pigmentation in affected individuals [38] [39] [40].

The transcription factor Nuclear Factor kappa-light-chain-enhancer of activated B cells (NF-κB) plays a crucial role in mediating inflammatory responses and cellular stress, making it an important factor in the pathogenesis of vitiligo. Its activation is influenced by redox status, linking oxidative stress to immune dysregulation in vitiligo. NF-κB is a key regulator of immune and inflammatory responses. Under normal conditions, NF-κB is sequestered in the cytoplasm by inhibitor proteins (IκBs). Upon activation by various stimuli, including oxidative stress, IκBs are phosphorylated and degraded, allowing NF-κB to translocate to the nucleus and activate target genes involved in inflammation and cell survival. In vitiligo, the activation of NF-κB can drive pro-inflammatory cytokine production, exacerbating autoimmune responses against melanocytes. ROS can activate NF-κB through multiple signalling pathways. Elevated ROS levels can modify cysteine residues in IκB proteins, leading to their degradation and subsequent NF-κB activation. Activation of NF-κB in response to oxidative stress can produce paradoxical effects on melanocyte survival. While NF-κB generally promotes cell survival by inducing antiapoptotic factors, excessive activation in chronic inflammation can also contribute to cell death through the upregulation of pro-apoptotic signalling. This dual role of NF-κB underscores its importance in the pathology of vitiligo.

NF-κB activation leads to the transcription of several pro-inflammatory cytokines, including TNF-α, IL-1, and IL-6. These cytokines can promote inflammation and recruit immune cells to the skin, further attacking melanocytes. Dysregulation of this pathway due to chronic oxidative stress creates a cycle of inflammation and immune-mediated damage, facilitating vitiligo progression. Targeting NF-κB signalling may offer therapeutic strategies for managing vitiligo. Inhibitors of NF-κB or agents that reduce oxidative stress could mitigate inflammation and protect melanocytes from autoimmune destruction [41] [42].

According to some studies, ROS generation in melanocyte mitochondria correlates with the vibrational properties of natural pheomelanin. The intimate physical interaction between mitochondria and melanosomes influences their viability through pigmentation [43]. Antioxidative enzymes are crucial in neutralising ROS and maintaining redox homeostasis in cells. Key antioxidative enzymes include: Super-oxide Dismutase (SOD), CAT, Glutathione Peroxidase (GPx) and Glutathione S-transferase (GST). SOD is pivotal in the first line of defence against oxidative stress. Studies show that reduced SOD activity in melanocytes leads to increased oxidative damage and contributes to melanocyte apoptosis. In patients with vitiligo, lower levels of SOD have been observed, correlating with disease severity. Catalase protects melanocytes from oxidative damage by breaking down hydrogen peroxide. Reduced CAT activity has been linked to increased levels of hydrogen peroxide in the skin, which can induce melanocyte apoptosis. Patients with vitiligo exhibit lower CAT activity, suggesting a compromised antioxidative defence [44] [45]. GPx is crucial in protecting cells from oxidative damage by reducing peroxides. Studies indicate that GPx activity is significantly lower in vitiligo-affected skin, leading to increased oxidative stress and further melanocyte damage. A meta-analysis revealed a strong correlation between vitiligo and low GPx levels. Out of the 23 studies that were looked into, patients from the Asian region had lower GPx than healthy controls [46]. GST are involved in detoxifying harmful substances and protecting cells from oxidative damage. Research suggests decreased GST activity in vitiligo patients may contribute to increased susceptibility to oxidative stress and melanocyte injury [47] [48].

Increasing evidence suggests that genetic factors, particularly SNPs in antioxidative enzyme genes, may play a critical role in the disease’s pathogenesis by influencing oxidative stress levels. SNP: rs4880 (*SOD2*) SNP is located in the mitochondrial manganese superoxide dismutase gene (*SOD2*). Variants such as rs4880 (Val16Ala) have been associated with altered enzyme activity and increased oxidative stress. Studies suggest that individuals carrying the Ala allele may have a higher risk of developing vitiligo due to reduced SOD2 activity. In contrast to the control, the CT and CTTT genotypes of the rs1041740 and rs17880135 variations were associated with vitiligo susceptibility. It has been demonstrated that the haplotypes InsCGTC and InsAGCC are risk factors for vitiligo susceptibility [49]. The SOD2 Thr58Ile (rs35289490) and Leu84Phe (rs11575993) polymorphisms were significantly associated with vitiligo patients, and the Val16Ala (rs4880) polymorphism was associated with active vitiligo patients [50]. Catalase (*CAT*) SNP: rs7943316 variants in the catalase gene have been associated with altered enzyme expression and activity. The SNP rs7943316 has been linked to reduced catalase activity, which can exacerbate oxidative stress in melanocytes and is thought to increase the risk of vitiligo [51]. Meta-analysis reported that 389 C/T polymorphisms in the *CAT* gene may be associated with vitiligo in Caucasians and other ethnicities [52] [53] [54].

AI has emerged as a powerful tool in enhancing the accuracy and efficiency of SNP diagnostics, offering novel insights into the genetic underpinnings of vitiligo and facilitating precision medicine approaches for diagnosis and treatment [55]. From genetic polymorphism diagnostics to personalised treat ment strategies, AI promises to improve the accuracy, efficiency, and effectiveness of vitiligo care. Regarding the integration of genomic data, AI-based algorithms can integrate vast quantities of data from diverse genomic sources, including whole-genome sequencing (WGS), targeted gene panels, and microarray data, to identify SNPs relevant to vitiligo. Preprocessing these datasets further may involve standardising genetic information, aligning sequences, and normalising expression data to ensure that variations are accurately identified. AI models can then be used to identify patterns in SNPs statistically associated with vitiligo susceptibility and progression. Detecting and classifying SNPs can be enhanced using AI algorithms based on corresponding genomic data. These models can distinguish between benign and pathogenic variants based on their frequency in healthy and disease populations, their position in functional genomic regions and their potential effects on gene function.

Furthermore, AI can be employed to predict the biological impact of identified SNPs, such as their potential role in immune regulation or melanocyte function, which are critical in the pathophysiology of vitiligo. AI can also analyse data from genome-wide association studies (GWAS) and other extensive cohort studies to identify SNPs located in immune-related genes, such as NLRP1, CTLA4, and HLA gene clusters, that may contribute to the autoimmune destruction of melanocytes. Furthermore, statistical analyses, such as clustering and principal component analysis (PCA), can uncover novel SNPs not previously implicated in vitiligo but may contribute to disease pathogenesis. Besides, the patients who exhibit variations in CTLA4 or NLRP1, which regulates inflammasome activity, can be identified early as having an increased risk of developing vitiligo [55] [56] [57]. As more genetic and clinical data become available, the role of AI in vitiligo diagnosis and therapy monitoring is expected to expand, potentially leading to more targeted interventions, reduced treatment failures, and better overall outcomes for patients.

### Role of AI in diagnostics, prediction and therapy monitoring of vitiligo

**Figure 2:**
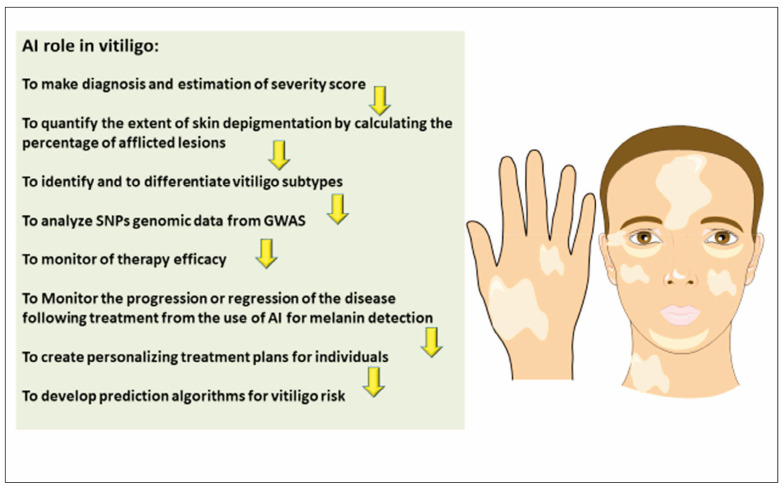
The role of AI in vitiligo diagnostics, monitoring and personalised treatment.

AI can substantially improve the efficacy of monitoring therapy and the personalisation of treatment plans for individuals with vitiligo (Figure 2). The AI-driven algorithms can analyse data from patient history, genetic polymorphisms, and treatment outcomes to predict the likelihood of a patient responding to specific therapies. By utilising historical treatment data and patient-specific characteristics, such as the presence of certain genetic markers or comorbidities, machine learning models can predict how patients will respond to topical treatments or systemic therapies. This information can be used to tailor treatments for each patient, maximising efficacy while minimising side effects. AI-powered tools can facilitate real-time monitoring of therapy responses by analysing clinical photographs, dermoscopic images, or even real-time patient-reported outcomes. Convolutional neural networks (CNNs) can be employed to assess changes in skin pigmentation, identify subtle alterations in depigmented lesions, and detect early signs of response or resistance to therapy.

Additionally, AI can help to monitor side effects and guide adjustments in treatment protocols. Another critical area where AI can contribute is in predicting disease relapse and long-term therapeutic outcomes. By leveraging longitudinal data, AI models can forecast the risk of relapse, enabling clinicians to modify treatment regimens preemptively. For instance, AI could identify patterns indicating when a patient is likely to experience a regression in repigmentation, allowing for more proactive and sustained intervention strategies. AI can integrate genetic, clinical, and phenotypic data to create personalised treatment plans for vitiligo patients. The AI tools can also help distinguish between vitiligo subtypes, enabling more accurate diagnosis and more precise categorisation of disease stages (e.g., active vs. stable vitiligo), which is critical for determining optimal therapeutic interventions. For instance, genetic variants associated with immune dysfunction could suggest that a patient may respond better to immunomodulatory therapies, such as topical calcineurin inhibitors, while melanocyte sur vival variants may indicate the need for phototherapy. Monitoring the progression or regression of the disease following treatment may benefit from using AI for melanin detection, which identifies and analyses melanin and dendritic cells. By incorporating genetic data, AI can help guide the decision-making process for clinicians, ensuring that each patient receives the most appropriate and effective treatment [58].

The clinical significance lies in the trans formative potential of AI-driven predictive algorithms in personalising medical treatment has been highlighted. The approach presented utilises AI to match patients with therapies tailored to their unique genetic makeup and disease presentation, offering a more precise and effective treatment plan. It can support dynamic treatment regimens that evolve in response to the patient’s shifting condition. This dynamic adaptability and integration of multi-source data can enhance early diagnosis and allow clinicians to make more informed, individualised treatment decisions. Continuous updating of treatment pro tocols is especially crucial for addressing complex, evolving diseases improving diagnostic accuracy and long-term patient outcomes. In doing so, AI-driven approaches have the potential to revolutionise clinical practice, providing healthcare professionals with the tools to make more timely and effective decisions.

## Conclusion and suggestions for future studies

In conclusion, the AI-driven predictive approach outlined here holds significant promise in improving personalised healthcare in vitiligo diagnostics and management. It offers a dynamic, evolving model of care that responds to patients’ individual needs, ensuring that treatments are consistently optimised. However, integrating such technology into clinical practice requires further validation and refinement. Future studies should focus on large-scale clinical trials to assess the effectiveness and scalability of AI algorithms in diverse patient populations. Additionally, research should explore integrating more complex data sets, including genetic diagnostics and environmental and lifestyle factors, to enhance the precision and adaptability of AI-based treatment systems. With continued advancements in AI technology and clinical integration, personalised medicine could become the standard of care, improving patient outcomes globally.

## Dodatak

### Acknowledgements

The work is part of the Horizon project approved by the European Research Executive Agency (REA) (Project 101159214 - ChatMED, Principal Investigator Prof Stevo Lukic, MD, PhD).

### Conflict of interest statement

All the authors declare that they have no conflict of interest in this work.
